# Abnormal Cerebrovascular Reactivity and Functional Connectivity Caused by White Matter Hyperintensity Contribute to Cognitive Decline

**DOI:** 10.3389/fnins.2022.807585

**Published:** 2022-03-04

**Authors:** Dan Yang, Ruomeng Qin, Lan Chu, Hengheng Xu, Ling Ni, Junyi Ma, Pengfei Shao, Lili Huang, Bing Zhang, Meijuan Zhang, Yun Xu

**Affiliations:** ^1^Department of Neurology, Drum Tower Hospital, Medical School and The State Key Laboratory of Pharmaceutical Biotechnology, Institute of Brain Science, Nanjing University, Nanjing, China; ^2^Department of Neurology, The Affiliated Hospital of Guizhou Medical University, Guiyang, China; ^3^Department of Radiology, Affiliated Drum Tower Hospital of Nanjing University Medical School, Nanjing, China; ^4^Jiangsu Key Laboratory of Molecular Medicine, Medical School of Nanjing University, Nanjing, China; ^5^Jiangsu Province Stroke Center for Diagnosis and Therapy, Nanjing, China; ^6^Nanjing Neurology Clinic Medical Center, Nanjing, China

**Keywords:** white matter hyperintensity, cognitive decline, cerebrovascular reactivity, functional connectivity, rs-fMRI

## Abstract

**Aims:**

This study aimed to investigate the relationships of impaired cerebrovascular reactivity (CVR) and abnormal functional connectivity (FC) with white matter hyperintensity (WMH)-related cognitive decline.

**Methods:**

A total of 233 WMH subjects were recruited and categorized into WMH-I (*n* = 106), WMH-II (*n* = 72), and WMH-III (*n* = 55) groups according to Fazekas visual rating scale. All participants underwent neuropsychological tests and multimodal MRI scans, including 3D-T1, and resting-state functional magnetic resonance imaging (rs-fMRI). The alterations of CVR maps and FC were further explored.

**Results:**

Subjects with a higher WMH burden displayed a lower CVR in the left medial occipital gyrus (MOG). The FC analysis using MOG as a seed revealed that the FC of the left insula, left inferior parietal lobule, and thalamus changed abnormally as WMH aggravated. After adjusting for age, gender, and education years, the serial mediation analysis revealed that periventricular white matter hyperintensity contributes indirectly to poorer Mini-Mental State Examination (MMSE) scores (indirect effect: β = −0.1248, 95% CI: −0.4689, −0188), poorer Montreal Cognitive Assessment (MoCA) (indirect effect: β = −0.1436, 95% CI: −0.4584, −0.0292) scores, and longer trail making tests A (TMT-A) (indirect effect: β = 0.1837, 95% CI: 0.0069, 0.8273) times, specifically due to the lower CVR of the left MOG and the higher FC of the left insula-MOG.

**Conclusion:**

The CVR decline of the left MOG and the abnormal FC of the left insula-MOG attributed to WMH progression were responsible for the poor general cognition (MMSE and MoCA) and information processing speed (TMT-A). The left MOG may act as a connection, which is involved in the processing of cognitive biases by connecting with the left insula-cortical regions in WMH individuals.

## Introduction

White matter hyperintensity (WMH), presented as hyperintensity of the subcortical white matter on T2-weighted image or fluid-attenuated inversion recovery (FLAIR) sequence, is frequently observed in the brain of elderly individuals (Alber et al., 2019). Epidemiologic studies revealed that WMH can be observed in 72–96% of European individuals over 60 years old (Longstreth et al., 1996) and 70% Chinese individuals over 50 years old (Han et al., 2018). Increasing evidences have revealed that the WMH burden exerts a negative impact on cognition, mainly in executive function and information process speed (Debette and Markus, 2010; Jiang et al., 2018; Alber et al., 2019). According to the Fazekas visual rating scales, WMH can be classified into four grades (grade 0, no WMH; grade 1, focal or punctate lesions; grade 2, beginning confluent lesions; and grade 3, confluent lesions) (Prins and Scheltens, 2015). Severe WMH is also closely related to incidence rates of stroke, dementia, and death (Debette and Markus, 2010; Prins and Scheltens, 2015). However, the underlying mechanisms by which increasing WMH burden triggers cognitive impairment and vascular events remain largely unknown. A better understanding of the mechanisms linking WMH with cognitive decline is of utmost importance.

Resting-state functional MRI (rs-fMRI) is a non-invasive imaging technique used to explore the aberrant intrinsic functional architecture of the brain. It can be used to calculate the temporal correlations among intrinsic fluctuations of blood-oxygen-level-dependent (BOLD) signals across functionally related areas, also known as functional connectivity (FC) (Damoiseaux et al., 2006). Additionally, rs-fMRI also offers a good option to obtain a quantitative mapping of cerebrovascular reactivity (CVR) (Golestani et al., 2016b; Taneja et al., 2019), which has been applied in several diseases, including carotid artery stenosis/occlusion, Moyamoya disease (De Vis et al., 2018; Ryan et al., 2018; Taneja et al., 2019), cerebral small vessel disease (CSVD) (Ni et al., 2021), and hypertension (Ma et al., 2021). CVR is the change of cerebral blood flow in response to a vasodilatory challenge and reflects underlying vascular dysfunction or pathology (Sobczyk et al., 2014). Impaired CVR is closely related to normal aging (Lu et al., 2011) and might be a sensitive predictor of dementia (Buratti et al., 2015). However, the underlying mechanisms by which a decrease in CVR triggers cognitive decline remain largely unknown. Additionally, previous studies have indicated that the altered FC and CVR in WMH individuals were associated with cognitive decline (Chen et al., 2019; Ye et al., 2019; Ni et al., 2021; Vipin et al., 2021). Nonetheless, none of these studies investigated the interrelationships among CVR and FC in WMH individuals, and their combined influence on WMH-related cognitive decline has never been examined. A prior study suggested that a change of CVR is significantly associated with FC in motor and executive control networks in healthy adults (Golestani et al., 2016a). Hence, in the present study, we aim to explore the effects of CVR and FC on WMH-related cognitive decline and the exact relationship among WMH, CVR, FC, and cognitive decline. We applied rs-fMRI to evaluate CVR maps and FC in WMH-I, WMH-II, and WMH-III populations. We investigated the relationships among WMH burdens, impaired CVR, FC, and cognitive decline in WMH individuals. We hypothesized that severe WMH may lead to local CVR decline, then induces abnormal FC, and finally results in cognitive decline.

## Materials and Methods

### Participants

The present research was conducted under the latest version of the Declaration of Helsinki and approved by the Nanjing Drum Tower Hospital Research Ethics Committee. All participants provided informed written consent before participating in the study. A total of 233 participants were consecutively recruited in Nanjing Drum Tower Hospital from January 2017 to April 2019. According to the Fazekas rating scale (Prins and Scheltens, 2015), the participants were divided into WMH-I (focal or punctate lesions, *n* = 106), WMH-II (beginning confluent lesions, *n* = 72), and WMH-III (confluent lesions, *n* = 55). WMH was categorized independently and unanimously by two radiologists, who visually evaluated the MRI without knowledge of the clinical profiles of the participants.

The inclusion criteria for WMH subjects were as follows: (Alber et al., 2019) age between 45 and 85 years old, Longstreth et al. (1996) the presence of mild to severe WMH on FLAIR, and (Han et al., 2018) no recent small subcortical infarctions (infarctions that presented a high signal in diffusion-weighted imaging were designated as a recent event). The exclusion criteria included the following: (Alber et al., 2019) a history of ischemic stroke with an infarct size larger than 1.5 cm in diameter or cardiogenic cerebral embolism, Longstreth et al. (1996) cerebral hemorrhage, Han et al. (2018) internal carotid artery or vertebral artery stenosis (>50%), Debette and Markus (2010) WMH due to immune-mediated demyelinating disease (multiple sclerosis, neuromyelitis optical, and acute disseminated encephalomyelitis), metabolic leukodystrophy, and genetic leukoencephalopathy, Jiang et al. (2018) cognitive impairments due to other neurological disorders, such as Alzheimer’s disease, Parkinson’s disease, and epilepsy (Prins and Scheltens, 2015) systemic diseases, such as cancer, shock, and anemia, Damoiseaux et al. (2006) prominent impairments of hearing or vision, and (Golestani et al., 2016b) MRI contraindications.

### Neuropsychological Examinations

The cognitive evaluations were completed by two professional neuropsychologists. The obtained raw scores were firstly converted to *Z*-scores. The Mini-Mental State Examination (MMSE) and the Montreal Cognitive Assessment (MoCA) were used to evaluate the global cognitive function. Other neuropsychological battery tests include Trail Making Tests A and B (TMT-A and TMT-B) and Stroop Color and Word Tests (SCWT) A, B, and C (SCWT-A, SCWT-B, and SCWT-C). In addition, both the Hamilton Depression Rating Scale and the Hamilton Anxiety Rating Scale were used to test the mental status of all subjects.

### Magnetic Resonance Imaging Scanning

The rs-fMRI, 3D-T1 weighted, and 3D-FLAIR images were extracted from all subjects using a 3.0-T magnetic resonance scanner (Philips Medical Systems, Netherlands). The imaging parameters used were as follows: high-resolution T1-weighted turbo gradient echo sequence: repetition time (TR) = 9.8 ms, echo time (TE) = 4.6 ms, flip angle (FA) = 8°, field of view (FOV) = 250 × 250 mm^2^, number of slices = 192, matrix sizes = 192 × 256 × 256, thickness = 1.0 mm; 3D-FLAIR images: TR = 4,500 ms, TE = 333 ms, FOV = 258 × 247 mm^2^, number of slices = 200, matrix sizes = 200 × 272 × 261; thickness = 1.0 mm. The rs-fMRI was acquired by a gradient-echo-planar imaging sequence: TR = 2,000 ms, TE = 30 ms, FA = 90°, FOV = 192 × 192 mm^2^, matrix size = 64 × 64, thickness = 4.0 mm, number of slices = 35; each functional image contained 240 volumes. During the scanning of rs-fMRI, the participants were instructed to keep their eyes closed, to stay as still as possible, and not to fall asleep or think about anything in particular. Additionally, T2-weighted and diffusion-weighted imaging sequences were collected to assess acute or sub-acute infarctions.

### Volume Quantification of Brain and White Matter Hyperintensity

The volumes of total brain, gray matter, and white matter were automatically obtained using Statistical Parametric Mapping (SPM8^1^) based on 3D-T1 images. The Wisconsin White Matter Hyperintensity Segmentation Toolbox^2^ was used to semiautomatically quantify total white matter hyperintensity (TWMH) volume, including deep WMH (DWMH) and periventricular WMH (PWMH) based on T1-weighted and FLAIR images.

### Evaluation of Cerebrovascular Reactivity

The CVR maps based on BOLD signals of rs-fMRI was calculated through SPM12 and in-house MATLAB (MathWorks, Natick, MA, United States) scripts. The preprocessing procedures included the following: (Alber et al., 2019) head motion correction, Longstreth et al. (1996) spatial smoothing through convolution with an isotropic Gaussian kernel of 8 mm, and (Han et al., 2018) linear detrending. Given that the global BOLD fluctuations in the frequency range of 0.02–0.04 Hz mostly contributed to natural variation in end-tidal (Et) CO_2_ levels (Liu et al., 2017), the rs-fMRI data were temporally filtered with a band-pass filter (filtering range: from 0.02 to 0.04 Hz). The average whole-brain rs-BOLD signal was calculated as the reference time course. A general linear model was employed in a voxel-wise manner, with the reference time course as the independent variable and the time course of each voxel as the dependent variable, generating a CVR index map. Then, the CVR index map was further normalized to the reference time course, yielding a relative CVR map. The relative CVR maps were spatially normalized to the standard Montreal Neurologic Institute (MNI) template, with a resampled voxel size of 3 × 3 × 3 mm^3^ so that a global voxel-wise between-subject comparison could be conducted.

### Functional Connectivity Analysis

The rs-fMRI was preprocessed using a toolbox for Data Processing and Analysis for Brain Imaging (DPABI) V2.3. The detailed preprocessing procedures were described in our previous study (Chen et al., 2019). Brain regions showing altered CVR among the three groups served as the seed to construct the resting-state FCs. Pearson cross-correlation analysis was applied between the seed time course and the time course of the whole brain voxels, and then Fisher’s *z*-transformation was used to improve the normality of the correlation coefficients *z* = 0.5 × ln[(1 + r)/(1 – r)]. Ultimately, the individual FC maps of each region showing a significant group difference of CVR were obtained.

### Statistical Analysis

White matter hyperintensity volumes were log-transformed to normalize the distribution. Differences in characteristics across the three groups were analyzed using one-way analysis of variance, chi-square (χ^2^) test, and Kruskal–Wallis test by SPSS 22.0 software (IBM Corp., Armonk, NY, United States). *P* < 0.05 was considered statistically significant.

A voxel-wise one-way analysis of covariance was performed on the comparison of CVR and FC maps among three groups using DPABI V2.3, adjusting for age, gender, and education years. The threshold was set at *P* < 0.001 and corrected by Gaussian random field (GRF) correction (voxel *p* threshold for the minimum cluster size < 0.001 and cluster *p* threshold < 0.001) for multiple comparisons. The number of minimum cluster voxels was 100. Subsequently, for the GRF-corrected statistically significant brain regions, a *post hoc* analysis was performed to investigate group differences between two arbitrary groups, additionally correcting with Bonferroni correction for multiple comparisons (*P* < 0.05/3 was considered statistically significant). Then, partial correlation analysis was conducted to assess the relationship of altered CVR and FC with clinical neuropsychological variables after adjusting for variables known to affect cognitive function (i.e., age, gender, and years of education).

Additionally, serial mediation analysis was performed to explore the mediating effect of altered CVR and FC on the relationship between WMH and cognitive scores after adjusting for age, gender, education years. The indirect effect of WMH on cognitive scores was estimated by multiplying the coefficients from three sequential linear regression models representing the pathway links (*a* × *b* × *c*). The models simultaneously estimate two shorter pathways, from WMH to altered CVR to cognitive scores (*a* × *b*_2_) and from WMH to abnormal FC to cognition scores (*a*_2_ × *c*), and the direct effect between WMH and cognitive scores adjusted for the indirect pathways (*d’* = *d* – *a* × *b* × *c* – *a* × *b*_2_ - *a*_2_ × *c*). We computed path coefficients and bias-corrected 95% confidence intervals for the size of the mediating effects with bootstrapping (*k* = 5,000 samples). The serial mediating effect is thought to be present if the 95% confidence interval (CI) does not contain zero. All the mediation analyses were conducted in PROCESS for the SPSS 22.0 framework.

## Results

### Demographic, Clinical, and Cognitive Data Among the Three Groups

The basic characteristics of the three groups are presented in Table 1. Among the three groups, there were no significant differences in all of the demographic and clinical factors (gender, education years, vascular risk factors, mental status, total brain volume, gray, and white matter volume, and bilateral hypothalamus volume; all *P* > 0.05) except for age and WMH volume (*P* < 0.05). We thus removed the effect of age in the following analyses. With the Fazekas score increased, the volume of WMH increased accordingly (*P* < 0.05). Compared with the WMH-I subjects, the WMH-III and WMH-II subjects exhibited poorer MoCA, SCWT-A, SCWT-B, and TMT-B (all *P* < 0.05). The WMH-III subjects displayed poorer MMSE, SCWT-C, and TMT-A in contrast with the WMH-I subjects (all *P* < 0.05).

**TABLE 1:** Basic characteristics and cognitive evaluations among the three groups.

Items	WMH-I (*n* = 106)	WMH-II (*n* = 72)	WMH-III (*n* = 55)	*F*/χ^2^	*P*-value
Age (years)	64.34 ± 6.85	66.78 ± 7.96	68.11 ± 6.47*^a^*	5.709	0.004*
Gender, male (% male)	55 (51.89)	37 (51.39)	24 (43.64)	1.093	0.579
Education (years)	11.42 ± 3.85	10.46 ± 3.87	10.24 ± 3.87	2.194	0.114
Hypertension, *n* (%)	65 (61.32)	53 (73.61)	37 (67.27)	2.926	0.231
Diabetes, *n* (%)	27 (25.47)	16 (22.22)	13 (23.63)	0.254	0.881
Smoking, *n* (%)	17 (16.04)	18 (25.00)	14 (25.45)	2.922	0.232
Hyperlipidemia, *n* (%)	20 (17.92)	20 (22.22)	11 (20.00)	2.141	0.343
WMH volume (ml)	1.94 ± 1.13	5.34 ± 1.86*^a^*	17.45 ± 10.44*^ab^*	93.88	<0.001*
Total brain volume (ml)	1,405.83 ± 116.18	1,410.40 ± 133.41	1,409.35 ± 135.44	0.03	0.974
Gray matter volume (ml)	572.42 ± 47.36	567.08 ± 50.51	551.45 ± 55.91	2.84	0.061
White matter volume (ml)	473.28 ± 46.79	477.23 ± 59.97	477.07 ± 58.24	0.13	0.88
HAMD	5.78 ± 4.62	6.00 ± 5.21	6.40 ± 4.92	0.270	0.764
HAMA	7.79 ± 6.51	9.32 ± 7.60	9.04 ± 7.50	1.141	0.321
***Z*-score of cognition**					
MMSE	0.20 ± 0.56	−0.09 ± 1.10	−0.27 ± 1.38*^a^*	4.418	0.013*
MoCA	0.45 ± 0.70	−0.26 ± 0.97*^a^*	−0.52 ± 1.19*^a^*	24.39	<0.001*
SCWT-A	−0.35 ± 0.47	0.11 ± 1.12*^a^*	0.51 ± 1.29*^a^*	14.26	<0.001*
SCWT-B	−0.25 ± 0.56	0.18 ± 1.41*^a^*	0.25 ± 0.96*^a^*	5.81	0.004*
SCWT-C	−0.24 ± 0.67	−0.08 ± 1.15	−0.35 ± 1.21*^a^*	6.08	0.003*
TMT-A	−0.23 ± 0.48	0.05 ± 1.15	0.38 ± 1.37*^a^*	6.83	0.001*
TMT-B	0.27 ± 0.97	0.14 ± 0.98*^a^*	0.35 ± 0.96*^a^*	6.71	0.002*

Values are presented as means ± standard deviation, median with minimum, and maximum or absolute numbers with percentages. HAMA, Hamilton Anxiety Rating Scale; HAMD, Hamilton Depression Rating Scale; MMSE, Mini-Mental State Examination; MoCA, Montreal Cognitive Assessment; SCWT, Stroop Color and Word Tests; TMT, Trail Making Test; WMH, white matter hyperintensity. *P < 0.05, statistical difference among the three groups. ^a^P < 0.05, statistically different from WMH-I. ^b^P < 0.05, statistically different from WMH-II.

### Cerebrovascular Reactivity Differences Among the Three Groups

**FIGURE 1:**
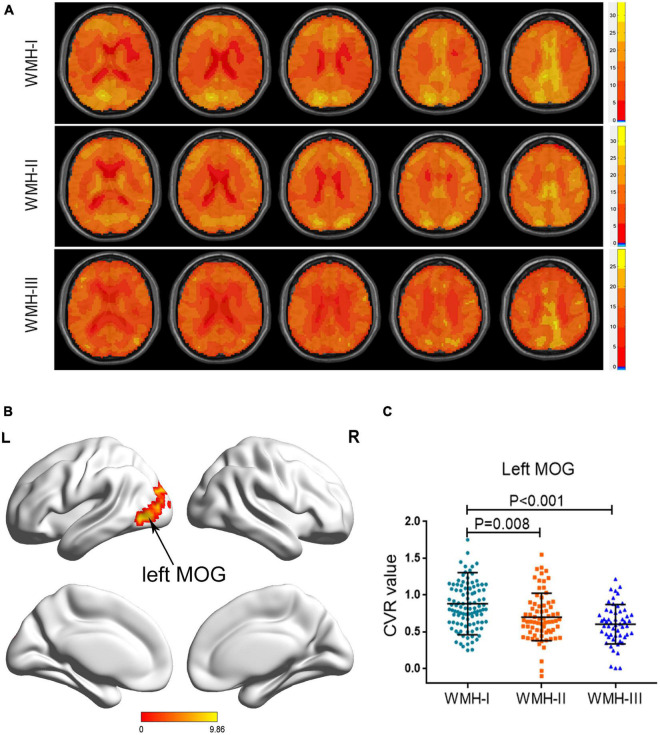
Group differences of CVR across the three groups. **(A)** Axial CVR mapping for each WMH cohort. **(B)** The brain areas displayed a significant difference among the three groups (left MOG). **(C)** The subjects in the WMH-II and WMH-III groups displayed a significantly lower CVR in the left MOG than the subjects in the WMH-I group. CVR, cerebrovascular reactivity; MOG, middle occipital gyrus; WMH, white matter hyperintensity.

Atlas maps of CVR based on the BOLD signal for each group are shown in Figure 1A. As shown in Table 2 and Figure 1B, the three groups displayed a significantly different CVR in the left middle occipital gyrus (MOG) after adjusting for age, gender, and education years with GRF correction for multiple comparisons. A *post hoc* analysis showed that there was a decreasing trend of CVR in the left MOG, accompanied by the increasing WMH grades. The WMH-II and WMH-III groups displayed a lower CVR in the left MOG than the WMH-I group (*P* = 0.008 in the WMH-II group and *P* < 0.001 in the WMH-III group vs. the WMH-I group, Bonferroni-corrected, Figure 1C). Relative to the WMH-II group, the WMH-III group showed a lower CVR in the left MOG. However, it did not achieve statistical significance.

**TABLE 2:** Brain region that displayed a different cerebrovascular reactivity among the three groups.

Brain areas	Peak MNI coordinates *x*, *y*, *z* (mm)	Peak *F* value	Number of cluster voxels
Left MOG	−30, −60, 21	10.488	285

*The voxel Z threshold < 0.001 with Gaussian random field correction (voxel p threshold for the minimum cluster size < 0.001 and cluster p threshold < 0.001) for multiple comparisons. MNI, Montreal Neurological Institute; MOG, middle occipital gyrus.*

### Functional Connectivity Differences Among the Three Groups

**FIGURE 2:**
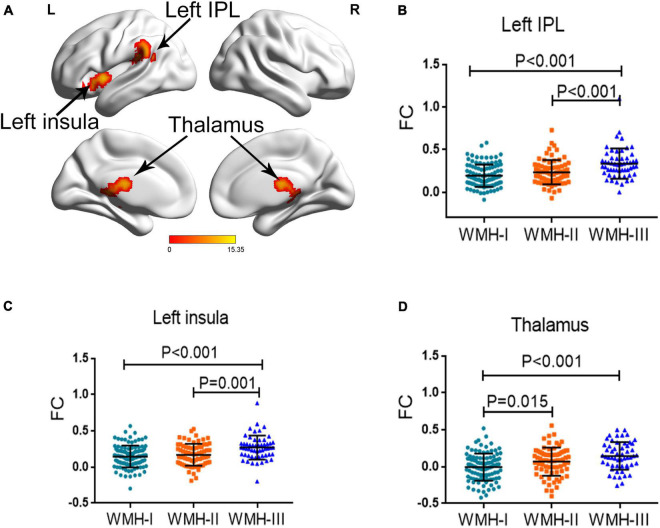
Group differences of FC across the three groups. **(A)** The regions displayed a significant difference among the three groups (left insula, left IPL, and thalamus). FC, functional connectivity; IPL, inferior parietal lobule. **(B)** The subjects in WMH-III displayed a significantly greater FC in the left IPL than the subjects in the other two groups. **(C)** The subjects in the WMH-III group displayed a significantly higher FC in the left insula than the subjects in the other two groups. **(D)** The subjects in the WMH-III and WMH-II groups displayed a significantly greater FC in the thalamus than the subjects in the WMH-I group. FC, functional connectivity; IPL, inferior parietal lobule; WMH, white matter hyperintensity.

Since the three WMH groups displayed a significant difference of CVR in the left MOG, we further investigated the differences of FC pattern among the three groups when using the left MOG as seed. As shown in Figure 2, the three groups showed a significantly different FC of the left insula, left inferior parietal lobule (IPL), and thalamus with the left MOG, after adjusting for age, gender, and education years with GRF correction for multiple comparisons. The detailed coordinate information of the above-mentioned regions is shown in Table 3. *Post hoc* analysis revealed that subjects in WMH-III displayed a significantly greater FC of the left IPL-MOG and the left insula-MOG than subjects in the other two groups (Figures 2B,C). In terms of FC of the thalamus-MOG, subjects in the WMH-III and WMH-II groups displayed a significantly greater FC in the thalamus than the subjects in the WMH-I group (Figure 2D).

**TABLE 3:** Brain areas that displayed different functional connectivity values among the three groups.

Brain areas	Peak MNI coordinates *x*, *y*, *z* (mm)	Peak *F* value	Number of cluster voxels
Left insula	−36, 6, 0	12.4497	116
Left IPL	−39, −39, 21	15.3541	252
Thalamus	−3, −18, 15	11.9213	252

*The voxel Z threshold < 0.001 with Gaussian random field correction (voxel p threshold for the minimum cluster size < 0.001 and cluster p threshold < 0.001) for multiple comparisons. MNI, Montreal Neurological Institute; IPL, inferior parietal lobule.*

### Correlations Among Altered Cerebrovascular Reactivity, Functional Connectivity, and Cognitive Performance

**FIGURE 3:**
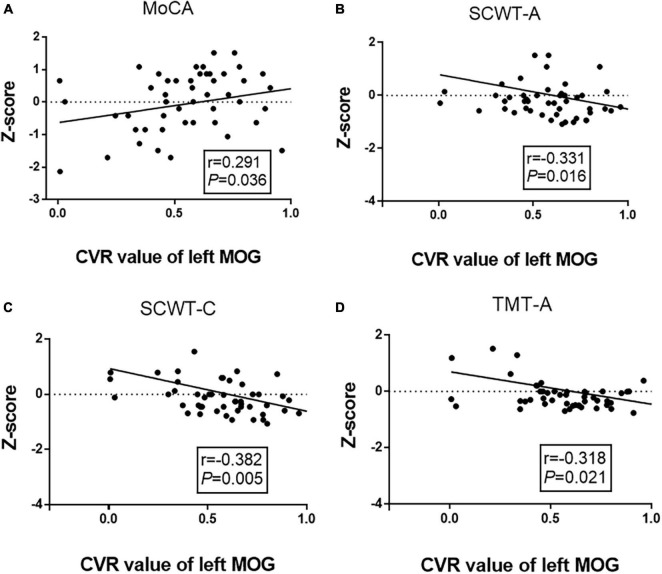
Correlation analyses between cognitive function and CVR of the left MOG in the white matter hyperintensity (WMH)-III group. **(A)** The CVR of the left MOG was positively correlated with MoCA score (*r* = 0.291, *P* = 0.036). **(B)** The CVR of the left MOG was negatively correlated with the time of SCWT-A (*r* = –0.331, *P* = 0.016). **(C)** The CVR of the left MOG was negatively correlated with the time of SCWT-C (*r* = –0.382, *P* = 0.005). **(D)** The CVR of the left MOG was negatively correlated with the time of TMT-A (*r* = –0.318, *P* = 0.021). The scores were z-transformed scores of the relevant tests. CVR, cerebrovascular reactivity; MOG, middle occipital gyrus; MoCA, Montreal Cognitive Assessment; SCWT, Stroop Color and Word Tests; TMT, Trail Making Test.

**FIGURE 4:**
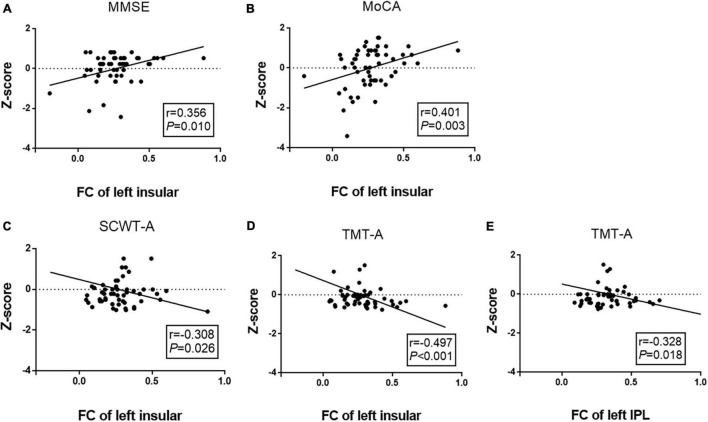
Correlation analyses between FCs and cognitive function in the white matter hyperintensity (WMH)-III group. **(A)** The FC of the left insula-MOG was positively correlated with MMSE score (*r* = 0.356, *P* = 0.010). **(B)** The FC of the left insula-MOG was positively correlated with the MoCA score (*r* = –0.401, *P* = 0.003). **(C)** The FC of the left insula-MOG was negatively correlated with the time of SCWT-A (*r* = –0.308, *P* = 0.026). **(D)** The FC of the left insula-MOG was negatively correlated with the time of TMT-A (*r* = –0.497, *P* < 0.001). **(E)** The FC of the left IPL-MOG was negatively correlated with the time of TMT-A (*r* = –0.328, *P* = 0.018). The scores were z-transformed scores of the relevant tests. FC, functional connectivity; IPL, inferior parietal lobule; MMSE, Mini-Mental State Examination; MoCA, Montreal Cognitive Assessment; SCWT, Stroop Color and Word Tests; TMT, Trail Making Test.

We performed a partial correlation analysis to explore the correlations among altered CVR, FC, and cognitive performance after adjusting for age, gender, and education years in the WMH-III group. As shown in Figure 3, the lower CVR in the left MOG was associated with a poor MoCA score (*r* = 0.291, *P* = 0.036, Figure 3A) as well as a longer SCWT-A (*r* = −0.331, *P* = 0.016, Figure 3B), SCWT-C (*r* = −0.382, *P* = 0.005, Figure 3C), and TMT-A time (*r* = −0.318, *P* = 0.005, Figure 3D). As shown in Figure 4, the FC of the left insula-MOG was positively associated with the MMSE score (*r* = 0.356, *P* = 0.010, Figure 4A) and the MoCA score (*r* = 0.401, *P* = 0.003, Figure 4B). The higher FC of the left insula-MOG was associated with a shorter SWCT-A (*r* = −0.308, *P* = 0.026, Figure 4C) and TMT-A time (*r* = −0.497, *P* < 0.001, Figure 4D). Similarly, the higher FC of the left IPL-MOG was associated with a shorter TMT-A time (*r* = −0.497, *P* = 0.018, Figure 4E). Nonetheless, there was no significant correlation of FC in the thalamus with cognitive performance. When analyzed the relationship between CVR and FC in the WMH-III group, we only found that the FC of the left insula was positively correlated with the CVR in the left MOG (*r* = 0.347, *p* = 0.012; the details are shown in Supplementary Table 1).

Furthermore, we performed a partial correlation analysis in the WMH-I and WMH-II groups, respectively. However, we just found that the CVR of the left MOG is negatively associated with the time of SCWT-C (*r* = −0.256, *p* = 0.008) in the WMH-I group (the detailed negative results of the partial correlation analysis in the WMH-I and WMH-II groups are shown in Supplementary Tables 1–3).

### Serial Mediating Effect of Abnormal Cerebrovascular Reactivity and Functional Connectivity on the Relationship Between White Matter Hyperintensity and Cognitive Performance

**FIGURE 5:**
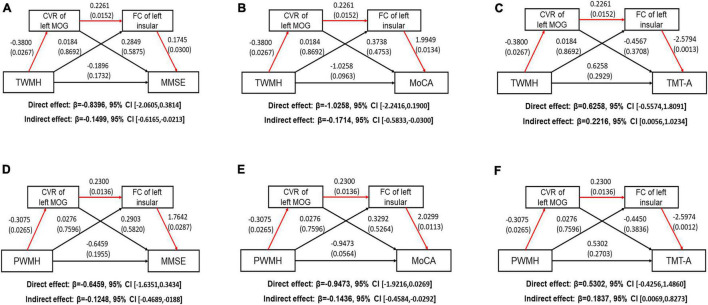
Path models show the direct and indirect relationships between white matter hyperintensity volume and cognitive function. The significant pathways are highlighted in red lines. For each connection, the standard coefficient and *P*-value are shown. The direct and indirect effect and their 95% CI are also shown. **(A)** TWMH volume was indirectly associated with poorer MMSE score through aberrant CVR of left MOG and FC of left insula-MOG (Indirect effect: −0.1499, 95% CI [−0.6165, −0.0213]. **(B)** TWMH volume was indirectly associated with poorer MoCA score through aberrant CVR of left MOG and FC of left insula-MOG (Indirect effect: −0.1714, 95% CI [−0.5833, −0.0300]. **(C)** TWMH volume was indirectly associated with longer TMT-A time through aberrant CVR of left MOG and FC of left insula-MOG (Indirect effect: −0.2218, 95% CI [0.0056, 1.0234]. **(D)** PWMH volume was indirectly associated with poorer MMSE score through aberrant CVR of left MOG and FC of left insula-MOG (Indirect effect: −0.1248, 95% CI [−0.4689, −0.0188]. **(E)** PWMH volume was indirectly associated with poorer MoCA score through aberrant CVR of left MOG and FC of left insula-MOG (Indirect effect: −0.1436, 95% CI [−0.4584, −0.0292]. **(F)** PWMH volume was indirectly associated with longer TMT-A time through aberrant CVR of left MOG and FC of left insula-MOG (Indirect effect: 0.1837, 95% CI [0.0069, 0.8273]. CI, confidence interval; CVR, cerebrovascular reactivity; FC, functional connectivity; MOG, middle occipital gyrus; TWMH, total white matter hyperintensity; MMSE, Mini-Mental State Examination; MoCA, Montreal Cognitive Assessment; TMT, Trail Making Test; PWMH, periventricular white matter hyperintensity.

We performed serial mediation analysis to explore the mediating effect of altered CVR and FC on the relationships between WMH and cognitive scores after adjusting for age, gender, and education years in the WMH-III group. We first found that the TWMH volume was indirectly associated with poorer MMSE score (indirect effect: −0.1499, 95% CI: −0.6165, −0.0213; Figure 5A) and MoCA scores (indirect effect: −0.1714, 95% CI: −0.5833, −0.0300; Figure 5B) and longer TMT-A time (indirect effect: β = 0.2216, 95% CI: 0.0056, 1.0234; Figure 5C), which was mediated serially by the aberrant CVR of the left MOG and FC of the left insula-MOG. When substituting the TWMH volumes with PWMH volumes or DWMH volumes in the mediation modal, we found that just the pathway of PWMH achieved significance, suggesting that the indirect effect of WMH on the above-mentioned cognitive scores was dominated by PWMH but not DWMH (the details are shown in Figures 5D–F).

Additionally, we also performed a *post hoc* mediation modal which hypothesized the WMH results from the CVR decline and then led to the FC change and cognitive decline in the WMH-III group. We did not find any mediation effect in this pathway (all 95% CI contained zero, Supplementary Table 4), suggesting that the mediation effect is specific to the WMH–CVR–FC–cognitive performance order.

## Discussion

In the present study, we demonstrated that, with the degree of WMH aggravated, the CVR of the left MOG decreased, and the FC of the left insula-MOG, left IPL-MOG, and thalamus-MOG abnormally changed. Notably, at the phase of severe WMH load (WMH-III), the aberrant CVR of the left MOG and FCs of the left insula/left IPL with MOG was associated with cognitive performance. Furthermore, PWMH contributes indirectly to poorer MMSE, MoCA, and TMT-A scores, specifically due to a lower CVR of the left MOG and a higher FC of the left insula-MOG, suggesting that severe WMH may lead to CVR decline, then induce abnormal FC, and finally result in cognitive decline. Additionally, this study provided a comprehensive understanding on the pathophysiology mechanisms of WMH progression and WMH-related cognitive decline.

The CVR patterns in WMH patients have been investigated by studies using transcranial doppler ultrasound or fMRI. Traditionally, the breath holding index of middle cerebral arteries *via* transcranial doppler ultrasound is useful for measuring global CVR, and previous researchers found that the global CVR decreased as the WMH aggravated (Bakker et al., 1999; Fu et al., 2006). Resting fMRI technique has been used to detect the regional CVR in CSVD (Ni et al., 2021) and hypertensive patients (Ma et al., 2021). Decreased CVR has been shown across different cortical regions, including the frontal cortex, parietal cortex, and occipital cortex in WMH patient (Ni et al., 2021). In the present study, we found that moderate–severe-WMH individuals displayed a lower CVR in the left MOG than mild-WMH individuals, and the decreased CVR was associated with general cognition (MoCA), information processing speed (SCWT-A and TMT-A), and executive function (SCWT-C) in severe-WMH individuals. The occipital lobe contains most of the anatomical region of the visual cortex and contributes to visual information processing and communication with the cerebral cortex as well as plays a crucial role in the perception of facial emotion. A previous study showed that the amplitude of occipital sources of resting-state alpha rhythms is related to Alzheimer’s disease neurodegeneration in the occipital lobe along with pathological aging (Babiloni et al., 2015). MOG plays an important role in category-selective attention modulating unconscious face/tool processing (Tu et al., 2013). Consequently, keeping in mind the above-mentioned overview, our finding may suggest that processing bias may be initiated as a perceptual visual bias, which may cause a series of cognitive decline.

Additionally, the FC patterns of the left insula-MOG, left IPL-MOG, and thalamus-MOG were abnormally increased when using the left MOG as seed. Increased FC might reflect an impaired balance between synaptic excitation and inhibition and the loss of the ability to inhibit activities. Furthermore, we found that the FC of the left insula-MOG was positively associated with general cognition (MMSE and MoCA) and information processing speed (SCWT-A and TMT-A). The FC of the left IPL-MOG was associated with information processing speed (TMT-A). The insula is instrumental in integrating disparate functional systems that are involved in cognition processing (Chang et al., 2013). It was reported that the function of the insula could be activated in a broad range of cognitive domains (Augustine, 1996; Shelley and Trimble, 2004). IPL is an important component of default mode network, which is known to be crucial in maintaining normal cognition (Buckner et al., 2008; Harrison et al., 2008). Consistent with these studies, our findings proved that the FC changes of the left insula and IPL were involved in WMH-related cognitive decline.

The causal relationships among WMH, CVR, FC, and cognitive functions remain unknown. We further explored this problem by multiple-step mediation analysis and confirm that severe WMH led to CVR decline of the left MOG, which then induced an abnormally higher FC between the left insula and MOG and finally resulted in cognitive decline. Conventionally, hemodynamic dysfunction, represented by CVR, is an etiological factor of WMH, which has been demonstrated by some animal model (Shibata et al., 2004; Ihara and Tomimoto, 2011) and clinical research (Sam et al., 2016a,b). However, mediation of the present study first revealed that severe WMH may lead to CVR decline in the left MOG, and the pathway just achieved significance in PWMH but not in DWMH. A previous study suggested that WMH can disrupt the left inferior fronto-occipital fasciculus, which is one of the connections of the left MOG (Chen et al., 2020). PWMH is more likely to disrupt the white matter microstructure than DWMH (Griffanti et al., 2018; Yang et al., 2020). From the view of the neurovascular unit, corresponding neuron cell body injury will be triggered after a severe axon injury (WMH-III), and then peripheral microvascular will be injured secondary, leading to CVR changes. Consistent with previous studies, we confirmed that severe PWMH is more likely to induce structural or functional disorders and then lead to cognitive decline than DWMH. Furthermore, our findings also demonstrated that the left MOG may act as a connection, which is involved in the processing of cognitive decline by being connected with the insular–cortical regions in severe WMH individuals.

The current study is innovative and acceptable for the following aspects: Firstly, rs-fMRI does not require any specialized cooperation, resulting in higher patient compliance and research accuracy. Secondly, the WMH subjects that we recruited in this work were relatively homogeneous and restricted to CSVD populations. Additionally, in the partial correlation and mediation analysis, we adjusted for all possible potential confounders, including age, gender, and education years.

Several limitations of the present work should be addressed. Firstly, no healthy controls were included in our study. A previous study using transcranial doppler ultrasound indicated that there was no significant difference in CVR between healthy controls and WMH-I groups (Bian et al., 2019). Future works should recruit healthy controls to verify whether the CVR of WMH-I subjects is lower than that of healthy controls. Secondly, we did not consider the impact of regional gray matter atrophy on FC and cognitive decline, which will have some influence on the generalization of the results. Finally, the sample is relatively small, and the nature of this study is cross-sectional. No causal inferences or directionality can be made. We are continuing to follow them up to validate our findings.

## Conclusion

The present study demonstrated that aberrant CVR decline in the left MOG and FC of the left insula with the left MOG attributed to WMH progression were responsible for the poor general cognition and information processing speed in severe-WMH individuals. Our findings may help to understand the mechanisms underlying the onset of cognitive decline in severe-WMH individuals and to explore novel brain markers of vascular cognitive impairment.

## Data Availability Statement

The original contributions presented in the study are included in the article/Supplementary Material, further inquiries can be directed to the corresponding authors.

## Ethics Statement

The studies involving human participants were reviewed and approved by the Nanjing Drum Tower Hospital Research Ethics Committee. The patients/participants provided their written informed consent to participate in this study.

## Author Contributions

YX designed this study. DY wrote the manuscript and statistically analyzed the data. RQ, LC, HX, and LN helped analyze MRI data. JM, PS, LH, and BZ helped collect data. YX and MZ revised the manuscript. All authors read and approved the final manuscript.

## Conflict of Interest

The authors declare that the research was conducted in the absence of any commercial or financial relationships that could be construed as a potential conflict of interest.

## Publisher’s Note

All claims expressed in this article are solely those of the authors and do not necessarily represent those of their affiliated organizations, or those of the publisher, the editors and the reviewers. Any product that may be evaluated in this article, or claim that may be made by its manufacturer, is not guaranteed or endorsed by the publisher.

## Footnotes

1
http://www.fil.ion.ucl.ac.uk/spm

2
https://sourceforge.net/projects/w2mhs

## Abbreviations

BOLD: blood-oxygen-level-dependent
CI: confidence interval
CSVD: cerebral small vessel diseases
CVR: cerebrovascular reactivity
DWMH: deep white matter hyperintensity
FC: functional connectivity
FLAIR: fluid-attenuated inversion recovery
GRF: Gaussian random field
IPL: inferior parietal lobule
MMSE: Mini-Mental State Examination
MRI: magnetic resonance imaging
MOG: middle occipital gyrus
MoCA: Montreal Cognitive Assessment
PWMH: periventricular white matter hyperintensity
rs-fMRI: resting-state functional magnetic resonance imaging
SCWT: Stroop Color and Word Tests
TMT: Trail Making Test
WMH: white matter hyperintensity.
