# Supplementary material for: Abnormal Cerebrovascular Reactivity and Functional Connectivity Caused by White Matter Hyperintensity Contribute to Cognitive Decline
Source: Front Neurosci. 2022 Mar 4;16:807585. doi: 10.3389/fnins.2022.807585 (PMC8930816; doi:10.3389/fnins.2022.807585)
Supplement: Supplementary file 1 [file Data_Sheet_1.PDF]

Supplementary material

Supplementary Table 1 correlations between altered CVR and FC in WMH-I, WMH-II, and WMH-III groups

Items	WMH-I	WMH-II	WMH-III
FC of left insula	0.076(0.176)	0.399(-0.103)	**0.012(0.037)**
FC of left IPL	0.158(0.140)	0.983(-0.003)	0.787(-0.038)
FC of thalamus	0.941(0.007)	0.249(-0.141)	0.117(0.220)

Abbreviations: FC, functional connectivity; IPL, inferior parietal lobule; WMH, white matter hyperintensity.
Values displayed were P value (correlation coefficient).

Supplementary Table 2 Correlations of altered CVR and FC with cognitive performance in WMH-I group

	CVR of left MOG	FC of left insula	FC of left IPL	FC of Thalamus
MMSE	0.535(0.062)	0.729(-0.035)	0.534(-0.062)	0.075(-0.176)
MoCA	0.262(0.112)	0.460(0.074)	0.791(-0.026)	0.876(-0.016)
TMT-A	0.833(-0.201)	0.513(0.066)	0.198(0.129)	0.260(0.113)
TMT-B	0.551(-0.061)	0.813(0.022)	0.429(0.081)	0.333(0.099)
SCWT-A	0.368(-0.092)	0.715(-0.037)	0.510(0.067)	0.296(0.106)
SCWT-B	0.330(-0.099)	0.809(-0.025)	0.819(0.023)	0.247(-0.118)
SCWT-C	**0.010(-0.256)**	0.601(-0.053)	0.891(-0.014)	0.238(-0.120)

Abbreviations: CVR, cerebrovascular reactivity; FC, Functional connectivity; IPL, inferior parietal lobule; MMSE, mini mental state examination; MoCA, the Montreal cognitive assessment; MOG, Middle occipital gyrus; SCWT, Stroop Color and Word Tests; TMT, trail making test.
Values displayed were P value (correlation coefficient).

Supplementary Table 3 Correlations of altered CVR and FC with cognitive performance in WMH-II group

	CVR of left MOG	FC of left insula	FC of left IPL	FC of Thalamus
MMSE	0.537(0.076)	0.078(-0.214)	0.781(-0.034)	0.969(0.005)
MoCA	0.779(0.034)	0.446(-0.093)	0.915(-0.013)	0.459(-0.091)
TMT-A	0.484(0.091)	0.151(0.185)	0.680(-0.054)	0.841(0.026)
TMT-B	0.273(0.149)	0.048(0.095)	0.527(0.086)	0.565(0.079)
SCWT-A	**0.049(0.251)**	0.984(0.003)	0.801(-0.032)	0.407(-0.107)
SCWT-B	0.576(0.073)	0.678(-0.054)	0.890(-0.018)	0.210(-0.163)
SCWT-C	0.619(0.065)	0.516(0.085)	0.458(0.098)	0.534(0.082)

Abbreviations: CVR, cerebrovascular reactivity; FC, Functional connectivity; IPL, inferior parietal lobule; MMSE, mini mental state examination; MoCA, the Montreal cognitive assessment; MOG, Middle occipital gyrus; SCWT, Stroop Color and Word

Tests; TMT, trail making test.
Values displayed were P value (correlation coefficient).

Supplementary Table 4 Serial mediation modal hypothesized CVR decline results from WMH and then leads to FC change and cognitive performance

Items	TWMH		PWMH		TWMH	
	Indirect effect	95% CI	Indirect effect	95% CI	Indirect effect	95% CI
MMSE	-0.0080	-0.1726,0.0715	-0.0150	-0.1840,0.0479	0.0049	-9.0116,0.1054
MoCA	-0.0091	-0.1879,0.0847	-0.0173	-0.1860,0.0625	0.0058	-0.0155,0.1085
TMT-A	0.0118	-0.1050,0.2952	0.0221	-0.0591,0.3303	-0.0074	-0.1849,0.0137

Abbreviations: CI, confidence interval; MMSE, mini mental state examination; MoCA, the Montreal cognitive assessment; TMT, trail making test.
